# Pleasure of paying when using mobile payment: Evidence from EEG studies

**DOI:** 10.3389/fpsyg.2022.1004068

**Published:** 2022-10-25

**Authors:** Manlin Wang, Aiqing Ling, Yijin He, Yulin Tan, Linanzi Zhang, Zeyu Chang, Qingguo Ma

**Affiliations:** ^1^School of Management, Zhejiang University, Hangzhou, China; ^2^Marketing Area, Michael Smurfit Graduate Business School, University College Dublin, Dublin, Ireland; ^3^School of Business Administration, Guizhou University of Finance and Economics, Guiyang, China; ^4^School of Electrical & Electronic Engineering, University College Dublin, Dublin, Ireland; ^5^Institute of Neural Management Sciences, Zhejiang University of Technology, Hangzhou, China

**Keywords:** mobile payment (m-payment), pain of paying, pleasure of paying, EEG, neuromarketing, consumer neuroscience, cashless payment

## Abstract

Mobile payment has emerged as a popular payment method in many countries. While much research has focused on the antecedents of mobile payment adoption, limited research has investigated the *consequences* of mobile payment usage relating to how it would influence consumer behaviors (e.g., purchase intention or willingness to pay). Here, we propose that mobile payment not just reduces the “pain of paying,” a traditional view explaining why cashless payment stimulates spending, but it also evokes the “pleasure of paying,” raising from the enhanced processing fluency in completing transactions. We tested this new conceptualization of “pleasure of paying” using EEG, complementing other behavioral measures. In two studies, we found that mobile payment effectively enhanced purchase likelihood (study 1, *N* = 66) and such an enhancement is generalizable to both hedonic and utilitarian products (study 2, *N* = 29). By employing EEG measures, we provided the first neural evidence of “pleasure of paying” in addition to the signal of “pain of paying.” Critically, we demonstrated that the “pleasure of paying” is a distinctive psychological mechanism that is induced by mobile payment usage and that the “pleasure of paying” joins the “pain of paying” to mediate the increased purchase intention. We discuss the contributions and implications of these results to the ongoing evolution of cashless payment societies.

## Introduction

Mobile payment^1^ (e.g., Alipay, Apple Pay, Amazon Pay, Google Pay) has been increasingly adopted by consumers and merchants in recent years. Survey research shows that mobile payment already beats all other payment methods in some East Asian countries (e.g., China, South Korea, and Vietnam) and the penetration rate of mobile payment in European countries such as Norway, Spain, and the United Kingdom has reached 20% (Buchholz, 2021). It is also forecasted that the global market value of mobile payment is going to triple from 1.7 trillion USD in 2021 to 6 trillion in 2027 (Yahoo, 2022). The rapid growth and bright prospect of mobile payment have drawn considerable attention from academia. Extant of prior research has focused on the understanding of key determinants catalyzing the mobile payment adoption (Zhou et al., 2010; Yang et al., 2012; Dahlberg et al., 2015; Oliveira et al., 2016; Bailey et al., 2017; Chao, 2019; Luna et al., 2019; Patil et al., 2020). Surprisingly, however, limited research has studied the *consequences* of using mobile payment on consumer behaviors such as the influences on willingness to pay (WTP), basket value, purchase intention, and satisfaction (Falk et al., 2016; Liu and Mattila, 2019; Boden et al., 2020; Liu and Dewitte, 2021; Ma et al., 2021b). This research contributes to the much-overlooked understanding of the impact of mobile payment on consumer behaviors by proposing and testing a novel mechanism: The pleasure of paying.

The neglect of investigating the impact of mobile payment usage may reflect an unwarranted assumption: Mobile payment is no different from other cashless payments such as bank cards. This assumption apparently makes sense for two reasons. First, considering its operational mechanism, mobile payment typically charges on an existing payment medium such as bank cards. Presumably, previous research findings on bank cards should be applicable to prescribe the impact of mobile payment on consumer behaviors (Feinberg, 1986; Raghubir and Srivastava, 2008; Thomas et al., 2011; Chatterjee and Rose, 2012; Shah et al., 2016). Second, considering its psychological mechanism, mobile payment is also low in transaction transparency similar to other cashless payments (Falk et al., 2016). Payment mediums that are low in transaction transparency would induce a weak perception of money paid and thus a reduction in the pain of paying (Zellermayer, 1996; Prelec and Loewenstein, 1998; Soman, 2003). This would suggest that, in principle, changes in consumer behaviors elicited by using mobile payment could have been well explained by the pain of paying effect.

However, results from recent studies have questioned whether the effects of previous cashless payments research, in particular, the pain of paying effect, simply and sufficiently extend to mobile payment. For example, Boden et al. (2020) showed that although mobile and credit card payments represent a similar extent of pain of paying, the mobile payment still led to a higher WTP than credit cards. In a meta-analysis study, Liu and Dewitte (2021) found no reliable evidence that the stated pain of paying mediated the impact of mobile payment on WTP or basket value as it did for credit cards. These results imply that mobile payment should be perceived as a distinctive mode of cashless payment and that there may be uncovered mechanisms, in addition to the pain of paying, that are specific to the usage of mobile payment.

We argue that mobile payment differs from bank cards, the most representative cashless payment method, in two important aspects. First, mobile payment is one of many functions embedded in a mobile device. Consumers use mobile devices for non-payment purposes such as taking photos, watching videos, checking social media, playing games and so on. These activities are pleasant and hedonic in nature, which may result in a positive perception of the mobile device as a payment medium (Ceravolo et al., 2019). In contrast, the payment function is almost the only function for bank cards (but also see Gafeeva et al., 2018 discussing how bank cards could integrate other functions such as loyalty programs). Little evidence indicates that people would perceive bank cards positively if they are not perceived as neutral or instrumentally (Durkin, 2000). Second, mobile payment is currently the fastest method to complete transactions in the field (Teo et al., 2015; Boden et al., 2020; Yan et al., 2021). In comparison to bank cards,^2^ mobile payment further removes required steps (or “ritual”) to complete transactions such as keying in the PIN or signing on the receipt.^3^ This enhanced processing fluency would elicit positive affect as evidenced by a multitude of psycho-physiological studies (Winkielman and Cacioppo, 2001; Reber et al., 2004; Winkielman et al., 2006; Alter and Oppenheimer, 2009, and see Schwarz et al., 2021 for a recent review).

Based on the above discussion, we propose that there would be a positive feeling raising from using mobile payment as “pleasure of paying.” Similar to the concept of pain of paying (Zellermayer, 1996), we suggest that the pleasure of paying is an immediate, integral emotion, which is derived and experienced from the act and the anticipated act of using the mobile payment for making purchases. Expectedly, it works in the opposite direction of the pain of paying: The pleasure of paying would soften the forgone ramifications of making a payment, rendering a motivational tendency to acquire goods or services. Indeed, much research has shown how being in a pleasant state, either transient or incidental, would trigger a higher inclination (or a lower resistance) for purchases or consumption (Fedorikhin and Patrick, 2010; Winterich and Haws, 2011; Schwarz and Clore, 2013; Achar et al., 2016; Bagozzi et al., 2016; Goenka and Van Osselaer, 2019; Poels and Dewitte, 2019). While proposing the pleasure of paying, we do not assume that mobile payment does not entail a reduction in the pain of paying because mobile payment, after all, is clearly qualified as a low transaction transparency payment method. The above theorization would lead to the following three specific hypotheses to be tested in this research:

*In addition to pain of paying there exists pleasure of paying when using mobile payment* such that:

H_1a_: *The usage of mobile payment would reduce pain of paying*

H_1b_: *The usage of mobile payment would enhance pleasure of paying*

H_2_: *Both pleasure of paying and pain of paying would facilitate consumer spending-related behaviours*

We tested the conceptualizations of pleasure of paying and pain of paying using the consumer neuroscience methodology of electroencephalogram (EEG). We focus on two event-related potentials (ERPs) to characterize the pain of paying and the pleasure of paying, respectively. N300, which is a negative deflection peaking at around 300 milliseconds (ms) after stimulus onset, has been associated with cognitive conflicts and incongruity toward evaluative targets (Zhang et al., 2011; Xue et al., 2013; Ma et al., 2016; Chang et al., 2018; Draschkow et al., 2018; Truman and Mudrik, 2018; Lu and Hou, 2020; Liu et al., 2021). This ERP index has been used in prior consumer research to signify a state of unpleasantness or irritation. For example, N300 was evoked when products did not match the stereotypical country-of-origin images of the products (e.g., watches from Belgium evoked a larger N300 than the Switzerland origin; Xie et al., 2018). N300 amplitude significantly increased when consumers considered the extended product categories were incompatible with the brand categories (e.g., a Coca-Cola branded television evoked a larger N300 than a Coca-Cola branded orange juice; Ma et al., 2007, 2021a). In the context of payment, we suggest that N300 is evoked due to the pain or the struggle when one has to forgo money in exchange for goods. Against H_1a_ and H_2_, we predict that the usage of mobile payment would decrease N300 amplitudes, and the reduction in N300 would promote spending-related behaviors.

The second ERP is the late positive potential (LPP), which is a slow, sustained positive ERP that manifests from 500 ms onward to several seconds (until the stimulus is offset). Decades of studies have found that changes in LPP amplitude are associated with the emotional significance of a stimulus, defined by the appetitive-aversive motivational tendency independent of arousal *per se* (Schupp et al., 2000; Hajcak et al., 2009; Weinberg and Hajcak, 2010; Gable and Harmon-Jones, 2013; Willroth et al., 2017; Hajcak and Foti, 2020). Research has shown that LPP is enlarged in responding to both pleasant and unpleasant emotionally evocative stimuli. This feature makes the LPP particularly suitable to validate the concept of pleasure of paying and also to differentiate it from the pain of paying. Specifically, if the use of mobile payment does *not* involve the pleasure of paying but only evokes the changes in pain of paying, we expect an enlarged but lower LPP amplitude for mobile payment vs. cash (i.e., a decrease in pain of paying). Alternatively, if the pleasure of paying does exist, we expect a *higher* LPP amplitude for mobile payment vs. cash (H_1b_), and increases in LPP should positively contribute to spending-related behaviors (H_2_).

In what follows, we report two experiments using EEG measures to test our hypotheses. We first describe how behaviorally the usage of mobile payment influences participants’ purchase intentions. Then, we analyze how ERP indicators vary as a function of payment methods. Subsequently, we perform mediation analyses to explore the mechanisms underlying the changes in purchase intentions and rule out alternatives. Finally, we discuss the contributions and managerial implications of this work, acknowledge the limitations and provide directions for future research.

## Study 1

### Materials and methods

#### Participants

Seventy-one right-handed native Chinese were recruited for the study. They were reimbursed 60 Chinese Yuan (CNY) for their participation. Participants declared that they had no history of neurological diseases or mental illnesses. Five participants were dropped due to technical issues to complete sufficient trials. This resulted in a final sample of 66 participants (20 females; M_age_ = 22.62, SD_age_ = 2.10) whose data was used for analyses. The study was approved by the Zhejiang University Ethical Review Board.

#### Purchase products

Forty headphones were used as the hypothetical purchase product. They were selected based on their real retail prices such that half of them were high-price headphones (M = 633.00 ± 370.88 CNY, min = 249.00 CNY, max = 1399.00 CNY) and the other half were low-price headphones (M = 43.20 ± 27.64 CNY, min = 9.90 CNY, max = 99.00 CNY). We took pictures of these headphones from a large Chinese e-commerce website. These pictures were gray-scale processed and adjusted to the same size (640 by 480 pixels) after removing brand logos using Adobe Photoshop.

#### Experimental procedure

Participants were comfortably seated in a room with sound and electrical insulation. A computer screen was positioned 1 m from their eyes at a visual angle of about 6.27°. We used Eprime 2.0 (Psychology Software Tools, Pittsburgh, PA, United States) to present a revised SHOP paradigm (Knutson et al., 2007, 2008; Karmarkar et al., 2014; Banker et al., 2021), with 2 (price levels: high vs. low) by 2 (payment methods: mobile vs. cash) within-subject design.

**Figure 1:**
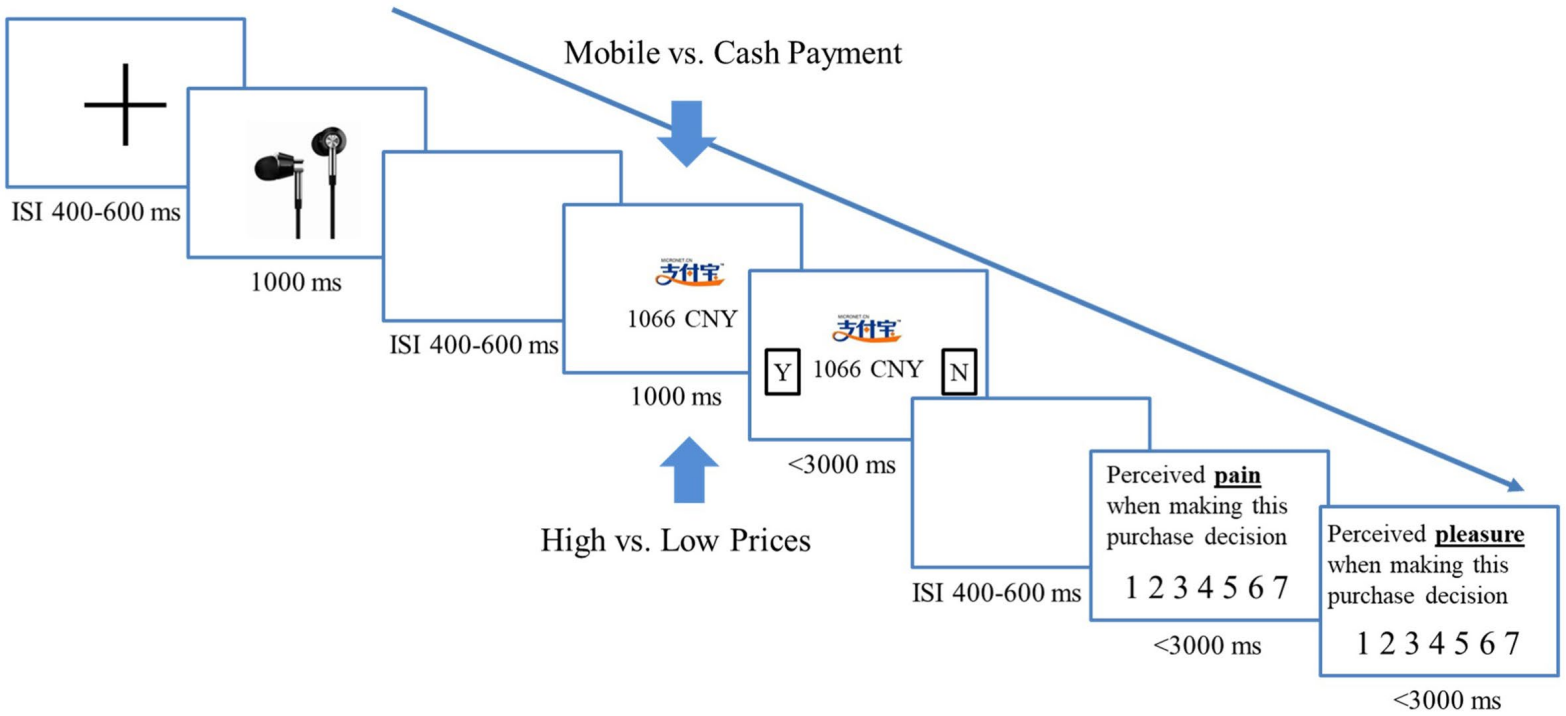
The experimental paradigm of study 1. For each trial, participants first saw the picture of the headphone and then were informed about the price (either high or low) of the headphone and the payment method to use (indicated by the Alipay icon or the CNY specimen). They pressed one of two keys to indicate whether they would like to buy this headphone. If they rejected the purchase, the next trial was advanced automatically. If they chose to purchase, they were further asked to rate the perceived pain and pleasure for making this decision on two separate 7-point scales.

Each trial began with a fixation cross, followed by showing a picture of the headphone and then a blank screen. Subsequently, the price of the headphone and the payment method to use were displayed. The payment method was indicated by either the Alipay icon (i.e., mobile payment) or the specimen of CNY (i.e., cash payment). Next, “Yes” and “No” checkboxes were shown on each side of the payment method, asking participants whether they would like to buy this headphone. Participants pressed one of the two keys on a keyboard to indicate their purchase intention. If participants rejected to purchase the headphone, the next trial was advanced automatically. If participants opted to buy the headphone, they were further asked to rate the perceived pain and pleasure of making this purchase decision on two separate screens using a 7-point scale. Larger numbers on the scale indicated a higher extent of pain or pleasure. Figure 1 depicts the sequences and the timing of each experimental event in detail.

Participants started with 20 practice trials before commencing 160 formal trials. To minimize body motions and mental fatigue from making decisions repeatedly, the formal trials were broken into four blocks of approximately 6 min with 5-min breaks between each block.

#### Electrophysiological recordings

EEG was used to record participants’ neural activity while they were performing the task. The EEG data and scalp voltage were captured (band pass 0.05–100 Hz, sampling rate 1,000 Hz) by Ag/AgCl electrodes implanted at 64 scalp locations using the extended international 10–20 system with a NeuroScan SynAmps 2 Amplifier (Scan4.5, Neurosoft Labs, Inc.). The left mastoid served as the reference electrode site, whereas the cephalic (forehead) served as the ground. A set of electrodes were inserted above and below the left eye to record the vertical electrooculogram (EOG), and the second pair was put at the left and right orbital rims to capture the horizontal EOG. When the data were recorded, all of the electrode impedances were required to be <5 kΩ.

### Data analyses

For behavioral data, we transformed the binary purchase decisions into purchase likelihood by dividing the number of purchase decisions by the total number of valid trials for each participant. We also computed the means of self-report pain and pleasure for each experimental condition and for each participant.

EEG data were pre-processed using NeuroScan 4.5. The data was re-referenced to the average of the left and right mastoids, corrected for electrooculographic artifacts due to eye movements using a regression-based algorithm and smoothed by using a 30 Hz low-pass filter (24 dB/octave; Semlitsch et al., 1986). Then, the pre-processed EEG data were time-locked, where −200 to 800 ms of the event of interest (i.e., when the headphone price and the payment method were shown) were extracted into 1,000 ms epochs, and the −200 ms pre-stimulus period served as the baseline for the whole epoch. We excluded trials if the amplifier chipping, bursts of electromyography activity, and peak-to-peak deflection exceed ±80 μV from the average (Haggard and Eimer, 1999; Mölle et al., 2002; Mantini et al., 2007). Finally, we averaged the data for each condition and for each participant. The data that were included in the subsequent analyses had a minimum of 30 sweeps (Mickleborough et al., 2014; Congedo et al., 2016; Roberts et al., 2021).

Based on previous research, we computed N300 by searching for a negative ERP peaking at around 280 ms with a time window of 20 ms on each side of this peak timing (i.e., the time window for N300 was from 260 to 300 ms in this study; Hamm et al., 2002; Franklin et al., 2007; Ma et al., 2016; Truman and Mudrik, 2018). LPP was computed based on a time window of 500–800 ms (Schupp et al., 2000; Weinberg and Hajcak, 2010). Time-locked EEG data at the frontal-central nine regions (F1, FZ, F2, FC1, FCZ, FC2, C1, CZ, C2) were extracted to compute N300 (Truman and Mudrik, 2018; Clark et al., 2019; Lu and Hou, 2020). Nine electrodes at the center-parietal regions (C1, CZ, C2, CP1, CPZ, CP2, P1, PZ, P2) were extracted to compute LPP (Choi et al., 2014; Morioka et al., 2016).

We employed multilevel mixed-effects regression to analyze the behavioral and ERP data. For multilevel mixed-effects regression, each experimental condition (i.e., four conditions for a 2 by 2 design) was treated as the fixed effect and was nested within each participant to cater for the random effects of participants’ responses. The model was estimated by the *mixed* function in Stata (StataMP 15.1) using a restricted-maximal likelihood approach. Contrast analyses were followed to examine simple effects for any significant main effects or interactions. We employed a set of mediation analyses to investigate the mechanisms that could explain the changes in purchase likelihood.

### Results and discussion

#### How did payment methods and prices influence participants’ purchase likelihood, and their perceived pain and pleasure?

We found a significant main effect of price levels on purchase likelihood (β = −0.141, *p* = 0.002, 95% BCI [−0.231–0.05]). Unsurprisingly, participants were less willing to buy high- vs. low-price headphones (M_high_price_ = 49.24% vs. M_low_price_ = 60.91%). In addition, we found a marginally significant interaction between price levels and payment methods (β = 0.087, *p* = 0.06, 95% BCI [−0.003 0.177]) such that in comparison to paying in cash, mobile payment effectively enhanced purchase intention for high-price headphones (M_high_price_mobile_ = 53.59% vs. M_high_price_cash_ = 44.88%). No significant effect was found for low-price headphones for different payment methods (M_low_price_mobile_ = 62.84% vs. M_low_price_cash_ = 58.97%, *p* = 0.404). These results indicated the advantage of mobile payment to facilitate purchases when making such decisions may require deliberation (e.g., buying expensive products).

Next, we analyzed participants’ self-report pain and pleasure as a function of price levels and payment methods. We only found a significant main effect of price on perceived pain (β = 2.922, *p* < 0.001, 95% BCI [2.70 3.13]) such that purchasing high-price headphones was perceived as more painful than the low-price ones (M_high_price_ = 4.755 vs. M_low_price_ = 1.876). The main effect of payment methods (*p* = 0.241) and its interaction with price levels (*p* = 0.577) were all insignificant. Similarly, we only found a significant main effect of price on perceived pleasure (β = 1.583, *p* < 0.001, 95% BCI [1.32 1.84]). Participants indicated a higher extent of pleasure for purchasing high- vs. low-price headphones (M_high_price_ = 5.248 vs. M_low_price_ = 3.591). The main effect of payment methods (*p* = 0.585) and the interaction term (*p* = 0.426) were insignificant. These results may imply a co-existence of pain and pleasure during purchase decisions that failed to be captured in previous research using bipolar scales.^4^

#### Mobile payment attenuated the ERP of N300 and enhanced the ERP of LPP

**Figure 2:**
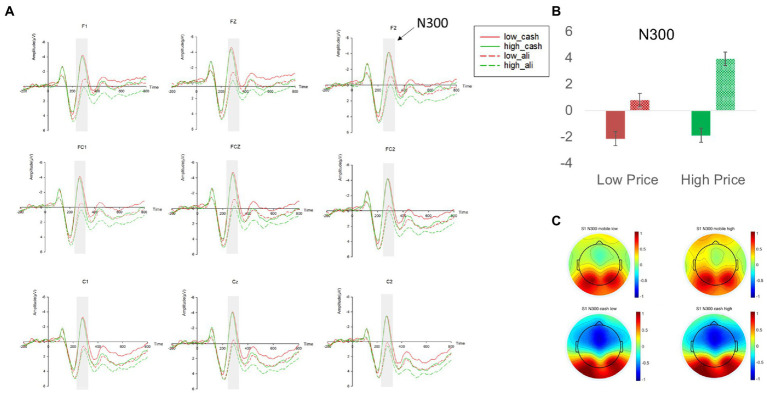
**(A)** The N300 amplitudes at nine electrodes (F1, Fz, F2, FC1, FCz, FC2, C1, Cz, C2) in study 1. **(B)** The mean N300 amplitudes across nine electrodes in different experimental conditions. **(C)** The topographies of the mean N300 amplitudes in different experimental conditions. Red and green in the line graphs and the bar graph represent low price and high price headphones, respectively; The dashed lines and the dashed filling in the bar graph represent the mobile payment condition, whereas the solid lines and the solid filling in the bar graph represent the cash payment condition; Error bars represent the standard errors. The average amplitude of N300 in respective experimental conditions is represented by a heat map with warm colors representing a lower amplitude of N300 and cold colors representing a higher amplitude of N300.

We first examined how N300, our index of pain of paying, was influenced by different price levels and payment methods. We found a significant main effect of payment methods (β = 3.544, *p* < 0.001, 95% BCI [2.792 4.296]). Contrast analyses revealed that in comparison to cash, mobile payment significantly reduced the amplitude of N300 for both high- and low-price headphones [M_mobile_high_price_ = 0.647 vs. M_cash_high_price_ = −3.453, χ^2^(1,66) = 114.19, *p* < 0.001; M_mobile_low_price_ = −0.121 vs. M_cash_low_price_ = −3.665, χ^2^(1,66) = 85.28, *p* < 0.001; see Figure 2 and Table 1 in Appendix]. The main effect of price levels (*p* = 0.58) and its interaction with payment methods (*p* = 0.305) were insignificant. This pattern confirmed H_1a_ that usage of mobile payment significantly attenuated the neural representation of pain of paying regardless of product prices.

**Figure 3:**
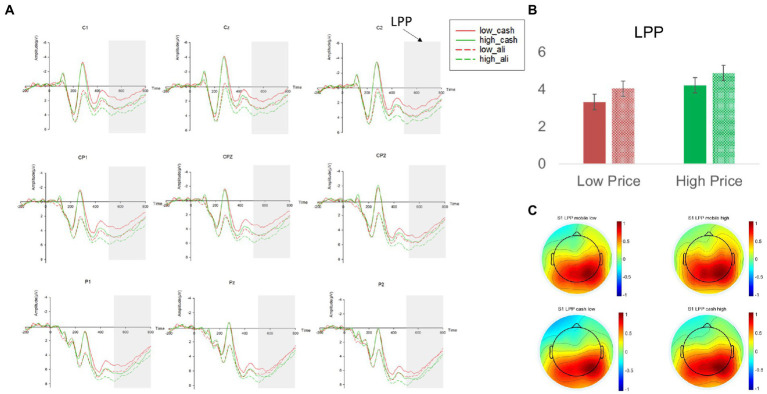
**(A)** The LPP amplitudes at nine electrodes (F1, Fz, F2, FC1, FCz, FC2, Cz, C2) in study 1. **(B)** The mean LPP amplitudes across nine electrodes in different experimental conditions. **(C)** The topographies of the mean LPP amplitudes in different experimental conditions. Red and green in the line graphs and the bar graph represent low price and high price headphones, respectively; The dashed lines and the dashed filling in the bar graph represent the mobile payment condition, whereas the solid lines and the solid filling in the bar graph represent the cash payment condition; Error bars represent the standard errors. The average amplitude of LPP in respective experimental conditions is represented by a heat map with cold colors representing a lower amplitude of LPP and warm colors representing a higher amplitude of LPP.

We then analyzed the changes in LPP amplitudes. We found a marginally significant main effect of payment methods (β = 0.715, *p* = 0.057, 95% BCI [−0.019 1.449]), suggesting that mobile payment evoked a *higher* LPP than cash (M_mobile_ = 4.467 vs. M_cash_ = 3.770; see Figure 3). As we indicated earlier, although LPP could index either a pleasant or an unpleasant state (Hajcak and Foti, 2020), it was not reasonable to interpret LPP as “pain of paying” in the current context because the pain would have been decreased for mobile payment usage. In other words, LPP magnitudes would have been lower for mobile payment than cash if LPP represents pain. The fact that LPP was *higher* for mobile payment than cash was congruent with H_1b_, suggesting that it represents a pleasant state. This result provided supporting evidence of the “pleasure of paying.”

In addition, we found a significant main effect of price levels on LPP (β = 0.894, *p* = 0.017, 95% BCI [0.159 1.629]). Compared to low-price headphones, deliberating decisions on purchasing high-price headphones evoked a significantly higher LPP (M_high_price_ = 4.547 vs. M_low_price_ = 3.681). Such an effect is also compatible with the interpretation that LPP represents a pleasant state. Previous literature has demonstrated how high price products (e.g., an expensive wine) could positively modulate consumption experience (e.g., the wine is tastier) at behavioral and neural levels (Plassmann et al., 2008). In the current context, a higher LPP toward high-priced headphones may reflect the anticipatory enjoyment from the purchases. The interaction between price levels and payment methods was not significant (*p* = 0.917).

#### Probing the mechanism underlying enhanced purchase likelihood induced by using mobile payment

While we found that the ERPs of N300 and LPP, representing pain of paying and pleasure of paying, decreased and increased respectively, as a result of using mobile payment, it is still a question of whether and how these two ERPs could explain the impact of payment methods on purchase likelihood (i.e., to test H_2_). We run a set of mediation analyses to address this question (using the bootstrap method with 5,000 repetitions).

The first mediation tested whether N300 alone, representing the pain of paying, was sufficient to explain enhanced purchase likelihood due to mobile payment usage. We found that the mediation path was insignificant (β = 0.007, 95% BCI [−0.021 0.036]). This result echoed Liu and Dewitte (2021)’s study finding no reliable evidence that the stated pain of paying mediated the impact of mobile payment on WTP or basket value. The second mediation tested whether LPP alone, representing the pleasure of paying, was an adequate mediator to explain enhanced purchase likelihood. We found that the mediation path was also insignificant (β = 0.008, 95% BCI [−0.0003 0.027]).

The third mediation model treated N300 and LPP as two sequential mediators, testing the hypothesis that both the feelings of pain of paying and pleasure of paying contributed to the changes in purchase likelihood. We found that the sequential mediation path was significant (β = 0.034, 95% BCI [0.015 0.074]) and the direct path was insignificant (β = 0.076, 95% BCI [−0.02 0.15]), suggesting a full mediation. Thus, in line with H_2_ this sequential mediation result indicated a joint impact of these two feelings in shaping participants’ purchase intention. To rule out the alternative, we ran a fourth mediation model testing whether N300 and LPP may compete with each other to influence the purchase likelihood. We found that neither the mediation of N300 (β = −0.009, 95% BCI [−0.039 0.021]) nor the mediation of LPP in this model was significant (β = 0.009, 95% BCI [−0.005 0.024]).

**Figure 4:**
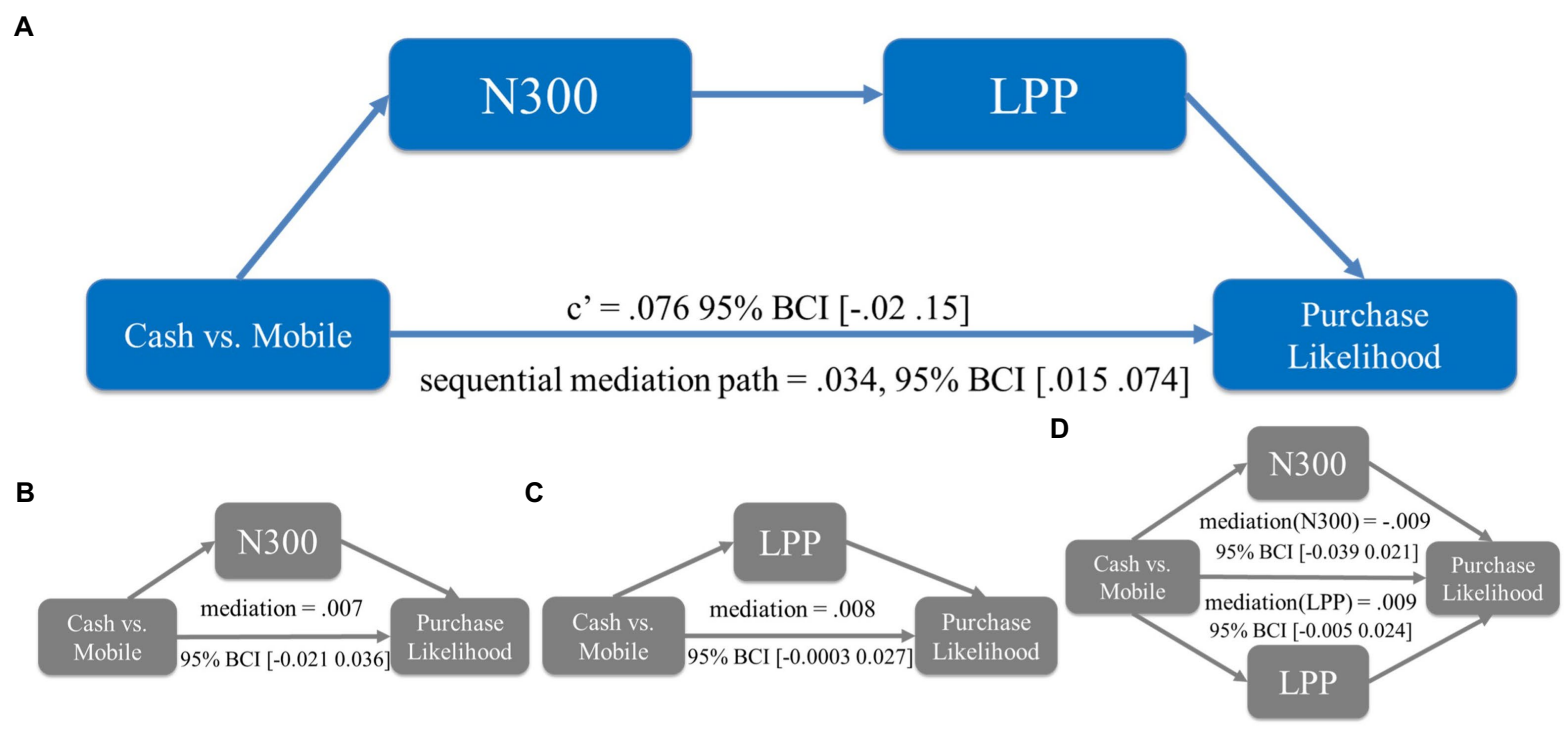
Mediation analyses in study 1. **(A)** The sequential mediation path was significant whereas the direct path was insignificant. **(B)** The mediation was insignificant when N300 was the single mediator. **(C)** The mediation was insignificant when LPP was the single mediator. **(D)** The mediation was insignificant when N300 and LPP were competing mediators. 95% bias-corrected confidence intervals (BCIs) from bootstrapping are represented in the brackets.

In summary, we found that the sequential mediation model was the only significant model among the mediation analyses that we conducted (see Figure 4). This result suggested that pleasure of paying is a unique psychological mechanism evoked by mobile payment usage in addition to the pain of paying, and that these two factors work together to explain the enhanced purchase likelihood.

#### A plausible cause of pleasure of paying: Enhanced processing fluency

We postulated that the pleasure of paying for mobile payment usage could be evoked due to its speedy processing in completing transactions. This speculation could be tested by analyzing the reaction times (RTs) when making purchase decisions. Supporting this speculation, we found a significant main effect of payment methods on RTs (β = −40.38, *p* = 0.008, 95% BCI [−70.31–10.46]) such that mobile payment was associated with shorter decision times than cash (M_mobile_ = 662.32 ms vs. M_cash_ = 702.40 ms). The main effect of price levels was marginally significant (β = 31.45, *p* = 0.073, 95% BCI [−2.91 65.83]) but the effect was in an anticipated trend: Participants spent more time deliberating on purchases for high price headphones than low price headphones (M_high_price_ = 698.82 ms vs. M_low_price_ = 666.99 ms). The interaction between price levels and payment methods was insignificant (*p* = 0.975). Therefore, we provided the evidence for a plausible cause of pleasure of paying that pleasure is derived from the fluency in completing transactions when using mobile payment.

## Study 2

In the previous literature, higher spending or higher consumption quantity, induced by paying with bank cards, was mostly found for products that were rich in hedonic features such as chocolates, snacks and unhealthy food (Soman, 2003; Thomas et al., 2011; Liu and Chou, 2020; Park et al., 2021). Headphones that were used as the purchase products in study 1 have blended characteristics of being functional (e.g., used for making phone calls) and hedonic (e.g., used to enjoy music). This raised the question of whether stimulation of purchase intention, induced by mobile payment in study 1, was limited to certain product types or was more generalizable.

This question motivated the design of study 2 in which we selected products that were prominent either in hedonic aspects or utilitarian aspects while their price levels were controlled. In addition, we would like to test whether the key findings in study 1 could be replicated.

### Materials and methods

#### Participants

Thirty-one right-handed naïve Chinese were recruited for this study. They satisfied the same participation criteria and they were paid 80 CNY for their participation. The sample size was determined based on the effect size of study 1 (Cohen’s *d* = 0.486). We estimated that a sample of 30 participants would achieve the statistical power at 0.82 with a type I error of 0.05. Two participants were excluded due to technical issues to complete the study. The data from the remaining 29 participants (11 females, M_age_ = 22.74, SD_age_ = 1.79) were used for statistical analyses. This study was reviewed and approved by the Zhejiang University Ethical Review Board.

#### Purchase products

We selected various products that were either rich in utilitarian features (e.g., printers, hairdryers, portable hard disks, power banks, and cabin bags) or hedonic features (e.g., small jewelry, perfumes, LEGO, chocolates, and Polaroid cameras). Crucially, the prices of these products in two categories were indifferent to each other [M_utilitarian_ = 228.07 vs. M_hedonic_ = 228.03, *t*(58) = 0.001, *p* = 0.999]. Similar to study 1, the pictures of these products were gray-scale processed and adjusted to the size of 640 by 480 pixels after removing brand logos.

#### Experimental procedure

The study followed an identical setting as in study 1. Eprime 2.0 (Psychology Software Tools, Pittsburgh, PA, United States) was used to present a revised SHOP paradigm (Knutson et al., 2007, 2008; Karmarkar et al., 2014; Banker et al., 2021) employed with a 2 (product categories: utilitarian vs. hedonic) by 2 (payment methods: mobile vs. cash) within-subject design.

**Figure 5:**
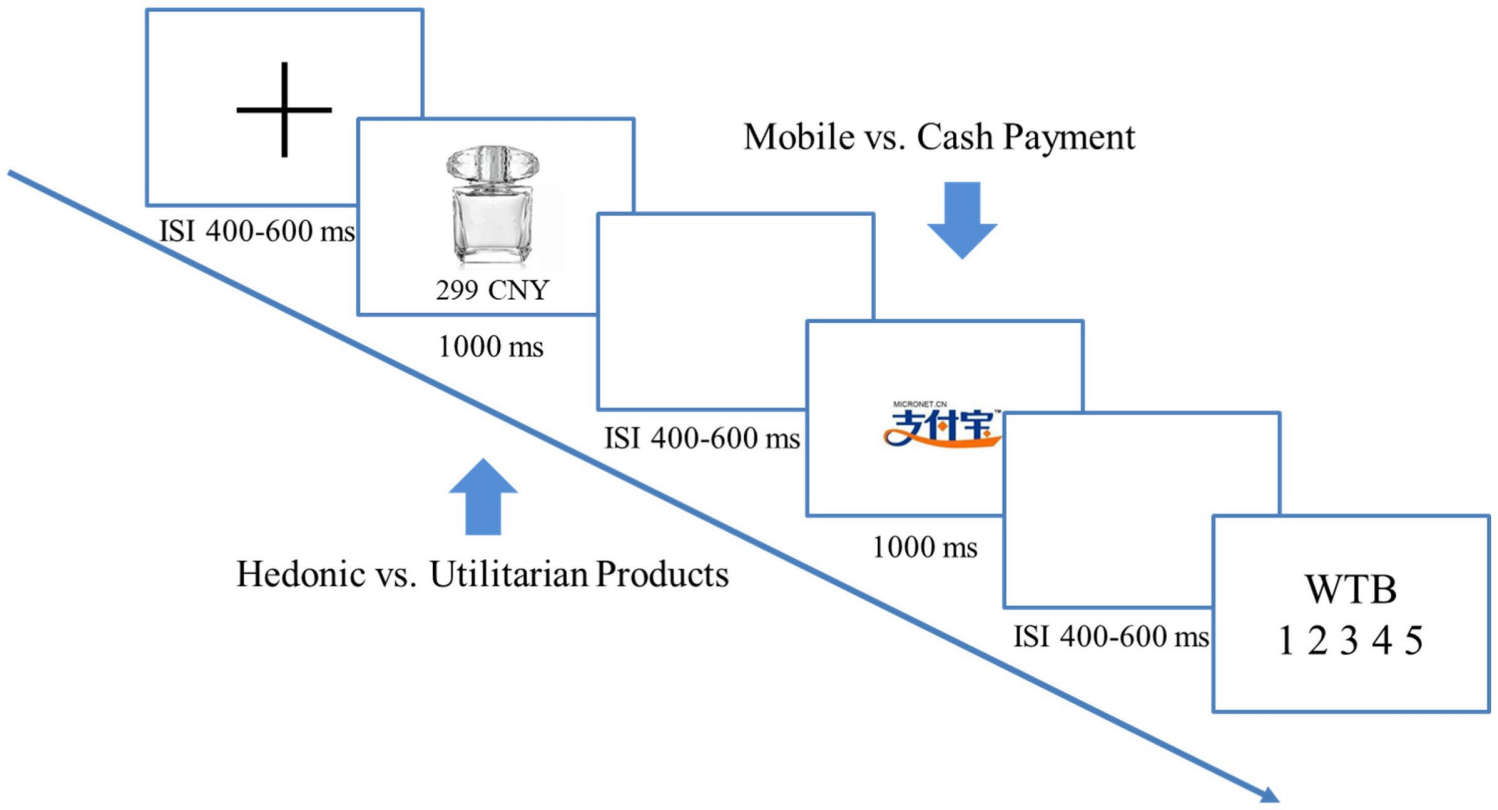
The experimental paradigm of study 2. For each trial, participants first found out the product (either hedonic or utilitarian) and its price and then were informed about the payment method to use (indicated by the Alipay icon or the CNY specimen). They were asked to indicate their willingness to buy (WTB) on a 5-point scale (higher numbers represent a higher WTB).

Each trial started with a fixation cross, followed by displaying a product, either a hedonic one or a utilitarian one and its price. After a brief blank screen, participants were prompted with the payment method for this trial, which was indicated by either the Alipay icon or the specimen of CNY. After another brief blank screen, participants were asked to rate their willingness to buy (WTB) of the product on a 5-point scale (1 = the least WTB, 5 = the highest WTB). EEG, with the same recording settings in study 1, was used to measure the neural activity throughout the experiment. Figure 5 depicts the sequences and the timing of each experimental event in detail.

### Data analyses

EEG data pre-processing was identical to study 1. The ERP of N300 was computed based on a negative peak at 300 ms with a 20 ms time window on each side of this peak timing (i.e., the time window for N300 was from 280 to 320 ms in this study) and LPP was computed for the time window of 500–800 ms.

Multilevel mixed-effects regression was employed to analyze the behavioral and ERP data. Although we had a relatively small sample size, each participant completed 240 trials to suffice statistical power. Given the experimental design, we treated each trial as a fixed effect and nested them within each participant to cater for the random effects in the multilevel regression. The same estimation procedures, contrast analyses and mediation analyses were used to test the statistical significance.

### Results and discussion

#### How did payment methods and product categories influence participants’ WTB and the ERPs of N300 and LPP?

For behavioral data, we found significant main effects of payment methods (β = 0.586, *p* < 0.001, 95% BCI [0.509 0.663]) and product categories (β = 0.102, *p* = 0.009, 95% BCI [0.025 0.179]). The interaction between these two factors was insignificant (*p* = 0.165). Contrast analyses on the main effect of payment methods showed that in comparison to cash, mobile payment significantly enhanced participants’ WTB for both hedonic [M_hedonic_mobile_ = 2.947 vs. M_hedonic_cash_ = 2.361, χ^2^(1,29) = 222.81, *p* < 0.001] and utilitarian [M_utilitarian_mobile_ = 3.127 vs. M_utilitarian_cash_ = 2.463, χ^2^(1,29) = 285.34, *p* < 0.001] products. Participants were more in favor of utilitarian products (M_utilitarian_ = 3.037 vs. M_hedonic_ = 2.412) despite that product prices in these two categories were similar to each other.

**Figure 6:**
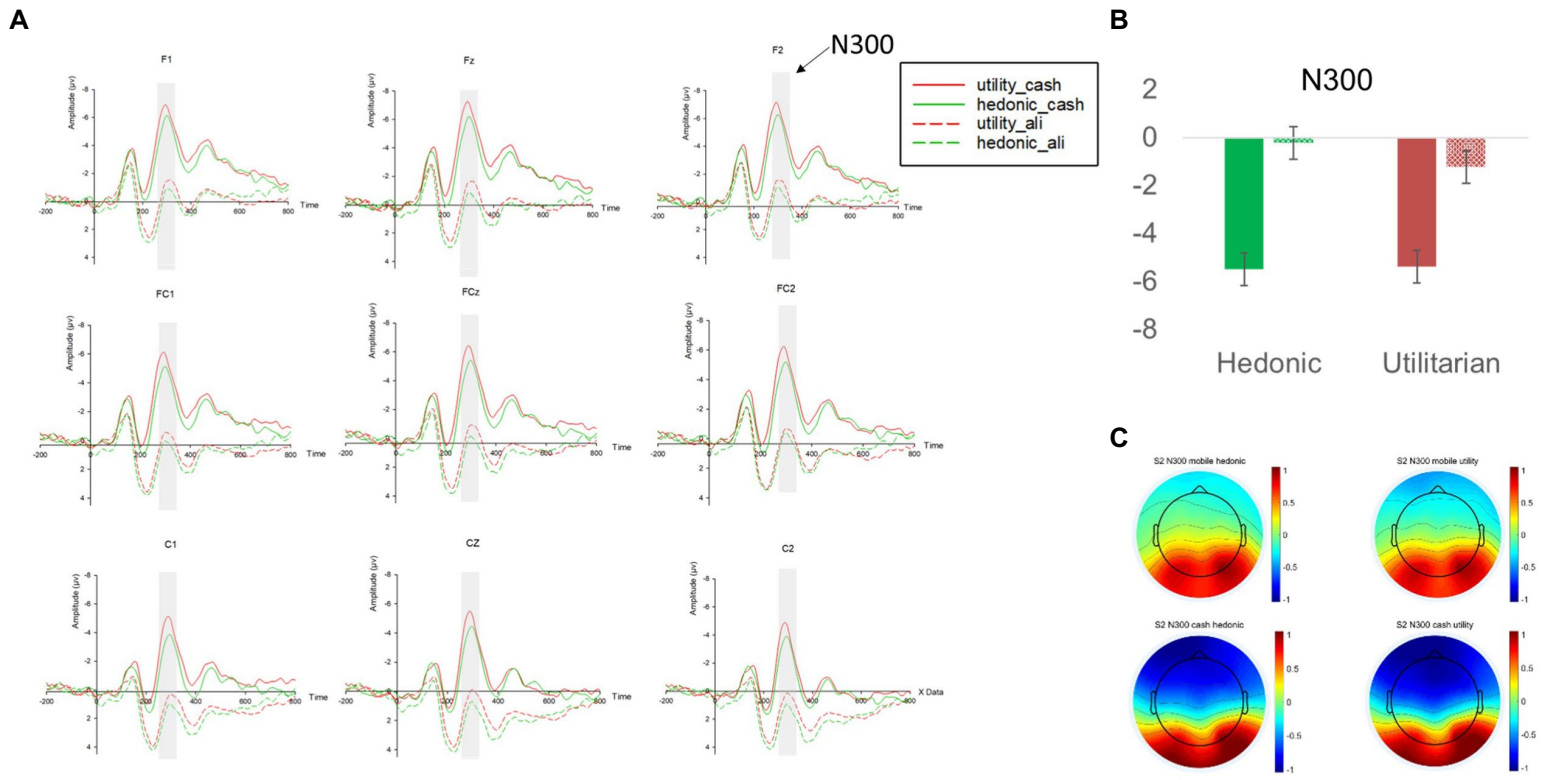
**(A)** The N300 amplitudes at nine electrodes (F1, Fz, F2, FC1, FCz, FC2, Cz, C2) in study 2. **(B)** The mean N300 amplitudes across nine electrodes in different experimental conditions. **(C)** The topographies of the mean N300 amplitudes in different experimental conditions. Red and green in the line graphs and the bar graph represent low price and high price headphones, respectively; The dashed lines and the dashed filling in the bar graph represent the mobile payment condition, whereas the solid lines and the solid filling in the bar graph represent the cash payment condition; Error bars represent the standard errors. The average amplitude of N300 in respective experimental conditions is represented by a heat map with warm colors representing a lower amplitude of N300 and cold colors representing a higher amplitude of N300.

Re-confirming H_1a_ we found a significant main effect of payment methods on N300 (β = 5.283, *p* < 0.001, 95% BCI [4.163 6.404]) suggesting that the usage of mobile payment reduced the neural representation of pain of paying. Contrast analyses revealed that in comparison to cash, mobile payment significantly decreased the amplitude of N300 for both hedonic [M_hedonic_mobile_ = −0.223 vs. M_hedonic_cash_ = −5.507, χ^2^(1,29) = 85.36, *p* < 0.001] and utilitarian products [M_utilitarian_mobile_ = −1.241 vs. M_utilitarian_cash_ = −5.398, χ^2^(1,29) = 52.86, *p* < 0.001; see Figure 6 and Table 2 in Appendix]. The main effect of product categories (*p* = 0.848) and its interaction with payment methods (*p* = 0.164) were insignificant.

**Figure 7:**
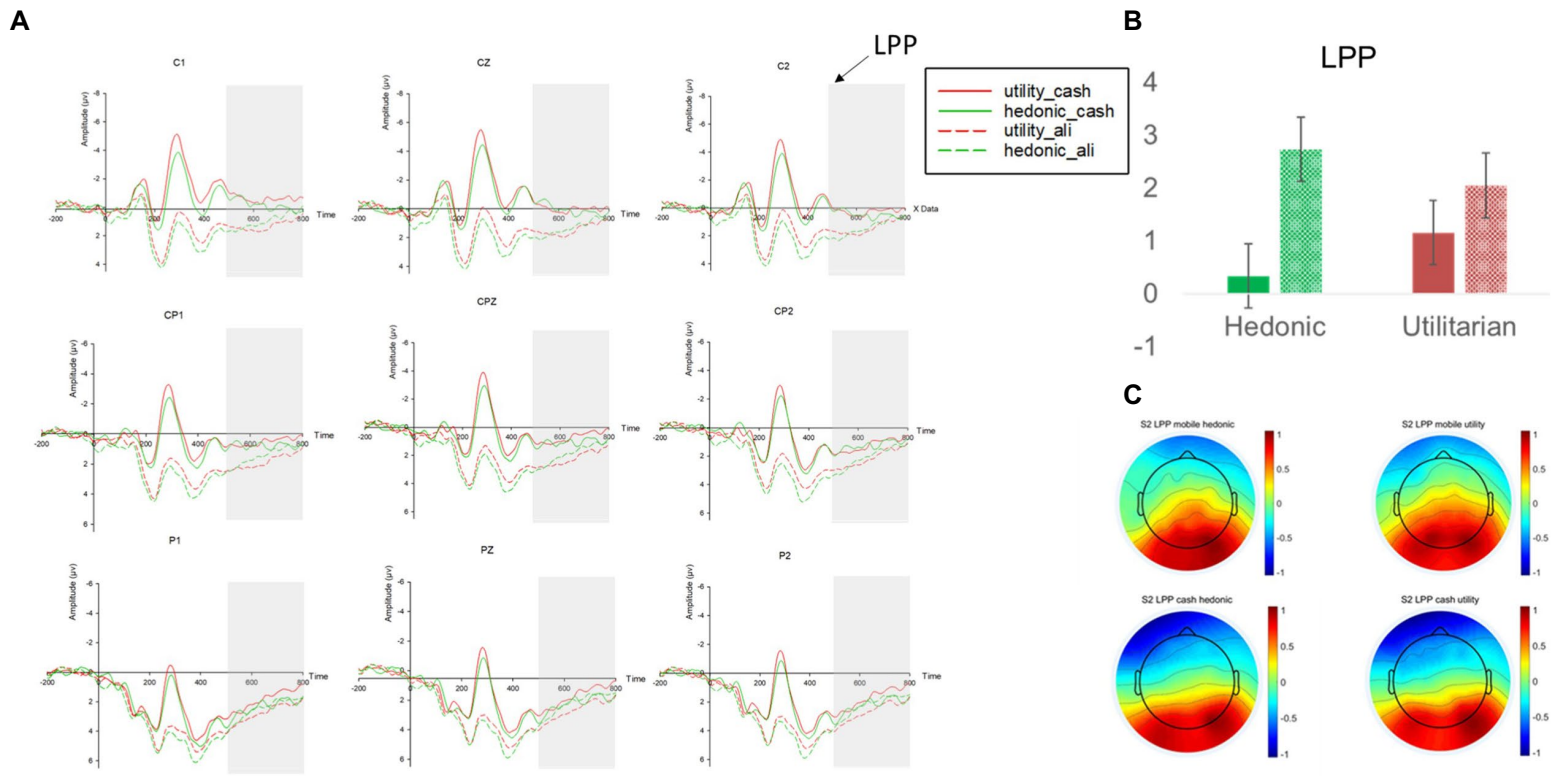
**(A)** The LPP amplitudes at nine electrodes (F1, Fz, F2, FC1, FCz, FC2, Cz, C2) in study 2. **(B)** The mean LPP amplitudes across nine electrodes in different experimental conditions. **(C)** The topographies of the mean LPP amplitudes in different experimental conditions. Red and green in the line graphs and the bar graph represent low price and high price headphones, respectively; The dashed lines and the dashed filling in the bar graph represent the mobile payment condition, whereas the solid lines and the solid filling in the bar graph represent the cash payment condition; Error bars represent the standard errors; The average amplitude of LPP in respective experimental conditions is represented by a heat map with cold colors representing a lower amplitude of LPP and warm colors representing a higher amplitude of LPP.

Similar patterns were found for the changes in LPP amplitudes (see Figure 7). Consistent with H_2b_, there was a significant main effect of payment methods (β = 2.402, *p* < 0.001, 95% BCI [1.081 3.725]). In comparison to cash payment, the usage of mobile payment evoked a significantly *higher* LPP amplitude for hedonic products [M_hedonic_mobile_ = 2.736 vs. M_hedonic_cash_ = 0.333, χ^2^(1,29) = 12.69, *p* < 0.001] but not for utilitarian products [M_utilitarian_mobile_ = 2.051 vs. M_utilitarian_cash_ = 1.154, χ^2^(1,29) = 1.77, *p* = 0.183]. The main effect of product categories (*p* = 0.224) and the interaction (*p* = 0.114) were all insignificant.

The above analyses indicated that, behaviorally, mobile payment facilitated participants’ WTB of products in different categories in comparison to cash payment. This payment effect was accompanied by changes in ERPs that we also observed in study 1: A decrease in N300, signaling a reduction in pain of paying (H_1a_) and an increase in LPP, reflecting an augment in pleasure of paying (H_1b_) when using mobile payment vs. cash.

#### N300 and LPP sequentially mediated the enhanced WTB induced by using mobile payment

**Figure 8:**
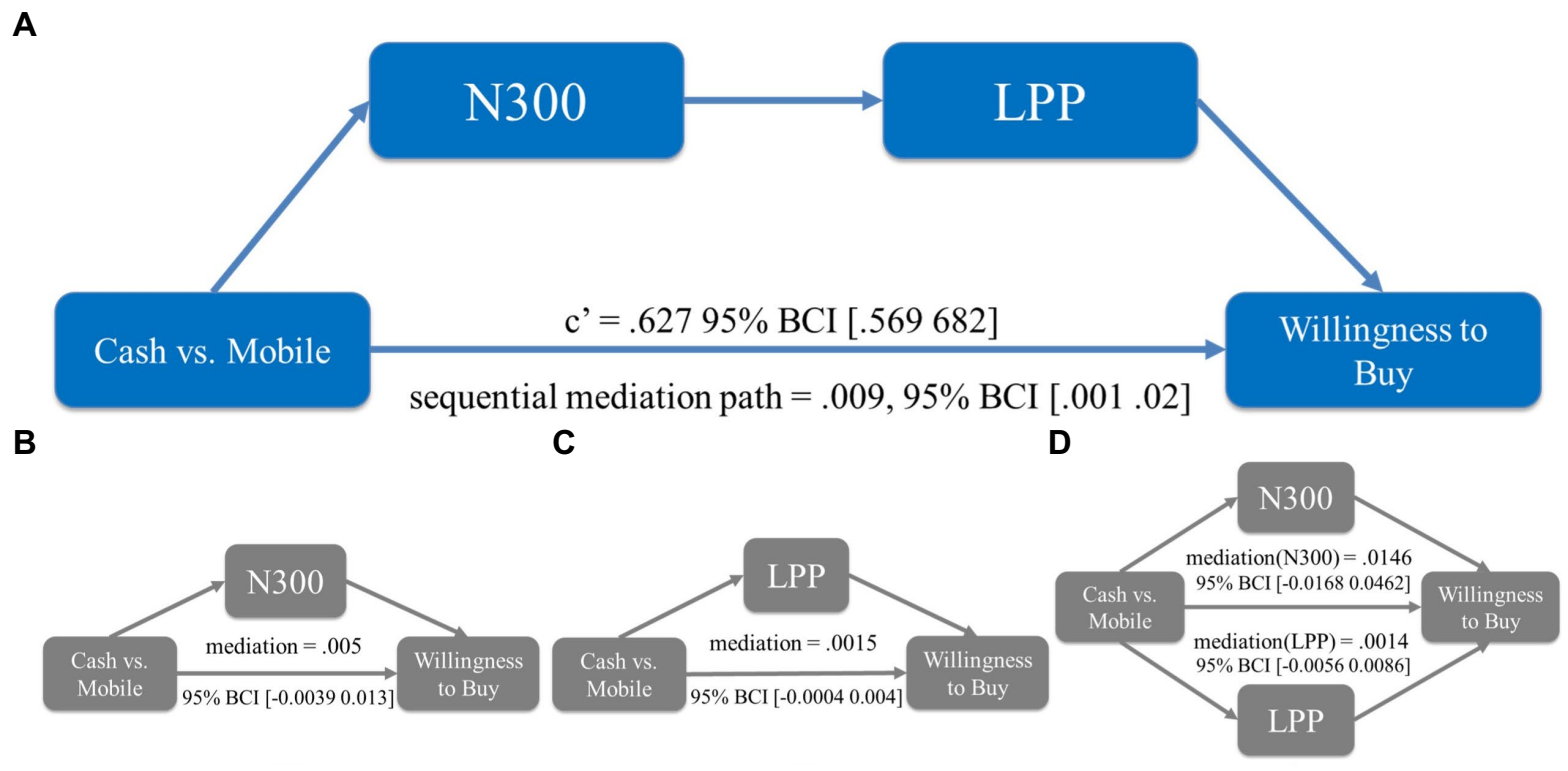
Mediation analyses in study 2. **(A)** The sequential mediation path was significant whereas the direct path was insignificant. **(B)** The mediation was insignificant when N300 was the single mediator. **(C)** The mediation was insignificant when LPP was the single mediator. **(D)** The mediation was insignificant when N300 and LPP were competing mediators. 95% bias-corrected confidence intervals (BCIs) from bootstrapping are represented in the brackets.

We conducted the same set of mediation analyses to test H_2_: Whether the enhanced WTB could be explained by the changes in the ERPs of N300 and LPP (see Figure 8). In two mediation analyses where N300 and LPP were treated as two single mediators, we found that mediation paths in respective models were insignificant (β_N300_ = 0.005, 95% BCI [−0.0039 0.013]; β_LPP_ = 0.0015, 95% BCI [−0.0004 0.004]). The parallel mediation model where N300 and LPP were constructed as competing mediators (β_N300_ = 0.0146, 95% BCI [−0.0168 0.0462]; β_LPP_ = 0.0014, 95% BCI [−0.0056 0.0086]) was also insignificant.

Strikingly, however, we found that N300 and LPP *jointly* mediated the enhanced WTB in a sequential mediation model (β = 0.009, 95% BCI [0.001 0.02]). The direct effect of this sequential mediation was also significant (β = 0.627, 95% BCI [0.569 0.682]), suggesting a partial mediation. Therefore, results from these mediation tests confirmed H_2_ and replicated what we found in study 1, demonstrating a unique involvement of two psychological processes, pain of paying and pleasure of paying, to influence participants’ WTB.

## General discussion

As a rapidly growing mode of cashless payment, mobile payment has sparked considerable interest to understand the drivers of its adoption (Dahlberg et al., 2015; Oliveira et al., 2016; Chao, 2019; Luna et al., 2019). While this research topic remains an important one, an equally important question that has been relatively overlooked is how mobile payment usage would influence consumer behaviors such as WTP, purchase intention, and consumer satisfaction. These indicators are important predictors of merchants’ revenues, which would in turn further drive the adoption of mobile payment because of merchants’ interest. This research contributes to the understanding of the *consequences* of using mobile payment against this background.

In two studies, we found that mobile payment, in comparison to cash payment, effectively enhanced purchase intention (e.g., purchase likelihood and WTB). Such an enhancement was found for both hedonic and utilitarian products, suggesting that stimulation of such purchase intention is more generalizable when using mobile payment than the bank cards (Soman, 2003; Thomas et al., 2011; Liu and Chou, 2020; Park et al., 2021). Critically, we theorized that the enhanced purchase intention is underpinned by the pleasure of paying, in addition to the traditional pain of paying effect. By using EEG, we captured neural signals indexing the pain of paying (i.e., N300) and the pleasure of paying (i.e., LPP) respectively. We found a decrease in N300 when using mobile payment vs. cash for both low- and high-priced products (study 1) and hedonic and utilitarian products (study 2). These results, as what would be predicted according to the cashless payment literature, confirmed H_1a_ that mobile payment would reduce pain of paying. More importantly, we provided the first evidence of pleasure of paying that was attested by the *increased* LPP for the usage of mobile payment vs. cash in both studies (i.e., support H_1b_). This pattern is in the opposite direction of N300, demonstrating that pleasure of paying is qualitatively different from pain of paying. Replicating prior research, we also found that the pain of paying *alone* was insufficient to mediate the enhanced purchase intention (Liu and Dewitte, 2021). Uniquely and robustly, we found that reduction in pain of paying (N300) and increase in pleasure of paying (LPP) *jointly* mediated the impact of mobile payment on enhanced purchase intention (H_2_). Other alternative mediation models were all insignificant. These results may explain Boden et al. (2020)’s finding that mobile payment out beats credit cards in eliciting a higher WTP despite that these two payment methods are similar in the extent of pain of paying. An overlooked mechanism at play is the pleasure of paying, which positively contributed to purchase intention. Taken together, this research extends the literature on cashless payment by characterizing a new psychological mechanism of pleasure of paying.

In addition to this theoretical contribution, this research also showcases how an interdisciplinary approach, combing EEG and traditional behavioral measures, could advance the understanding of psychological substrates underneath cashless payment. In the past decade, marketing and consumer research has witnessed an increasing application of cognitive neuroscience methodologies such as functional magnetic resonance imaging (fMRI), EEG, and eye-tracker among others (Ling and Plassmann, 2020). These methodologies circumvent self-report hurdles when the designated psychological constructs to study are implicit, swift and difficult to articulate (Plassmann et al., 2015; Karmarkar and Plassmann, 2019). A few recent research has employed these methodologies to study cashless payment and provided some unique insights (Mazar et al., 2016; Banker et al., 2021; Park et al., 2021). For example, despite that the term “pain of paying” has been widely cited and acknowledged, it is often considered metaphorically rather than literally because it is inferred from self-report measures based on bipolar scales. Using fMRI, Mazar et al. (2016) demonstrated that the pain of payment is tangible, which was reflected in brain regions processing affective pain. In this research, we employed EEG which is superior in temporal resolution to capture neural activity. This methodology provided us with a fine-grained portrayal of emergence and changes in pain of paying and pleasure of paying over the temporal evolvement. In another study using fMRI, Banker et al. (2021) found that credit card (vs. cash) purchases evoked a significant neural activation in the striatum and ventromedial prefrontal cortex, two brain regions that are consistently activated for receiving rewards. This result hinted at a rewarding experience when paying with credit cards. Our studies extended this work by demonstrating that pleasantness is also evoked by using mobile payment. All this evidence from these pioneering studies using cognitive neuroscience methods provides researchers with new angles to ruminate on the new mechanisms behind cashless payments.

## Limitations and future research

This research suffers from several limitations to be acknowledged. First, the participants of our studies were young adults from a developed city in China who are accustomed to using mobile payment. It is unclear how much the pleasure of paying is presented among a more representative population (e.g., different age groups or professions). It would also be interesting to explore whether the extent of using mobile payment would influence the pleasure of paying. While one may expect that only habitual users would exhibit pleasure of paying (as demonstrated in our samples), it is equally likely that new adopters would also derive the pleasure of paying due to the significant convenience of making payments that they experience.

Second, although we characterized the pleasure of paying, we are yet to fully understand the causes of such pleasure. We reasoned that one of the causes is the enhanced processing fluency for making transactions when using mobile payment. In study 1, we found that mobile payment was associated with significantly shorter RTs in comparison to cash payment. This provides the initial evidence to support that enhanced processing fluency is a possible cause of pleasure of paying. There are other plausible causes for the pleasure of paying. For instance, the pleasure of paying could be derived from the positive perception of the mobile device due to its embedded entertainment functions (Ceravolo et al., 2019). We call for future research to test this and other tenable causes that may generate the pleasure of paying.

Third, we identified the pleasure of paying in our studies by benchmarking mobile payment vs. cash payment. One might question whether the pleasure of paying would still hold when the alternative payment is bank cards or POS independent mobile payment such as PayPal. We conjecture that pleasure of paying is not confined by payment methods *per se* but to what extent the payment method is linked to the causes to evoke pleasure. In recent years, touch payment has been implemented as a new function for some bank cards. Transactions are made by touching the card with a POS device without requesting a PIN. This payment function also facilitates transaction fluency and, in principle, it would also evoke the pleasure of paying. However, since touch payment is limited to small value purchases (e.g., up to 50 EUR in many European Union countries), it is unclear whether the pleasure of paying might be derived from this new function of bank cards. We suggest that future research could compare different forms of cashless payments to investigate how they differ in terms of the extent of pleasure of paying.

Fourth, this research is yet to test the moderators of the pleasure of paying. Since we demonstrated that processing fluency is a cause of pleasure of paying when using mobile payment, we postulate that the extent of pleasure would be further enhanced if an individual is under a high cognitive load. This is because processing fluency would be much more appreciated when one’s cognitive resources are constrained. Future research could include cognitive load as a potential moderator. Finally, future research could extend our findings by investigating whether and how mobile payment would also influence transaction utility (i.e., how much consumers perceive the value of a deal) or post-consumption utility (e.g., customer attachment or loyalty to a product after consumption). Prior research found opposite effects on these two utilities for the usage of credit cards. While the usage of credit cards could shift consumers’ focus on product benefits and thus would enhance the perceived value of products (Chatterjee and Rose, 2012), the post-consumption attachment to the products would be attenuated because of a weak sense of commitment during the transaction process when using credit cards (Shah et al., 2016). We suggest that mobile payment may work differently on the post-consumption utility: The pleasure of paying could be misattributed to strengthen the product attachment.

## Managerial implications

While previous research focused on how customers’ experience could be promoted from the design, display and acquisition of the products or services, the present research demonstrated that the payment method is an overlooked contributing factor. Indeed, payment methods could play an important role in influencing the customer journey (Lemon and Verhoef, 2016). A more convenient, flexible, and time-saving way of paying would improve the customer experience. Subsequently, it would promote the quality and the trust of the relationship between customers and retailers (Taylor, 2016; Briedis et al., 2020; Sun et al., 2021). In this regard, our finding bears significant managerial implications for providers and retailers to improve and embrace mobile payment. To mobile payment providers, since the positive experience could be derived from the fluency of completing transactions, they could further enhance the fluency and handiness of paying across different payment formats (e.g., face-recognition payment, wearable payments). To retailers, they should realize that mobile payment is not a neutral instrument for getting the transaction done, but it may be used to facilitate customer experience and adhesiveness. Some retailers are already a step ahead. For instance, residents of the Disney Resort can use MagicBand, a bracelet mobile device, to pay for merchandize. From the perspective of pleasure of paying, this would effectively enhance customers’ Disney experiences. Retailers could also take advantage of this smart technology by broadening their sales channels through mobile payments. For example, they could make mobile checkout an option in all sales channels and reward points to encourage adoption and usage. Finally, while multiple parties may enjoy the ongoing evolution of cashless payment societies, care should be taken to account for the security of using such services. This is particularly impending since the usage of mobile devices is convolved with individual lives. Whether and how much mobile payment is allowed to access personal data should be closely supervised by regulatory bodies.

## Data availability statement

The raw data supporting the conclusions of this article will be made available by the authors, without undue reservation.

## Ethics statement

The studies involving human participants were reviewed and approved by Zhejiang University Ethical Review Board. The patients/participants provided their written informed consent to participate in this study.

## Author contributions

MW and QM conceived this research. MW, YH, YT, and LZ conducted the experiments under the supervision of QM. MW, AL, and ZC analyzed the data. MW and AL drafted the manuscript. All authors contributed to the article and approved the submitted version.

## Funding

This work was supported by grant no. 72062008 and grant no. 71942004 from the National Natural Science Foundation of China. This work was also supported by National Research Fund of China (Nos. AWS14J011). The work was also funded by China Scholarship Council to Manlin Wang.

## Conflict of interest

The authors declare that the research was conducted in the absence of any commercial or financial relationships that could be construed as a potential conflict of interest.

## Publisher’s note

All claims expressed in this article are solely those of the authors and do not necessarily represent those of their affiliated organizations, or those of the publisher, the editors and the reviewers. Any product that may be evaluated in this article, or claim that may be made by its manufacturer, is not guaranteed or endorsed by the publisher.

## Footnotes

^1^There are two broad categories of mobile payments: (1) transactions made based on near-field communication (NFC) technology which is commonly used at point-of-sales (POS) with handheld devices; (2) transactions made based on wireless communication technology such as PayPal, which could be used independent from POS. This research refers to mobile payment as the NFC-based technology.

^2^We note that some bank cards are now equipped with a touch payment function for small value purchases. We discuss how this new function of bank cards is related to mobile payment in the General Discussion.

^3^Even if the mobile payment requests an identity check for large value purchases, it is typically verified by fingerprints or facial features of the account holder. This process could be done instantly by tapping the thumb or looking at the screen.

^4^Bipolar scales, with one end marks pain and the other end marks pleasure, have been widely used in previous cashless payment research to characterize pain of paying (Zellermayer, 1996; Casagrande et al., 1997; Soman, 2001; Khan, 2011; Soster et al., 2014; Shah et al., 2016; Park et al., 2021). However, these scales might not be able to capture pain- and pleasure-of paying independently.

## Appendix

TABLE 1 The results of multilevel regressions in Study 1.

**Table 1:**

Study 1			
Dependent Variable	N300	LPP	Purchase Intention
Payment Method (0 = cash, 1 = mobile payment)	3.544*** (0.383)	0.715^†^ (0.375)	0.039 (0.046)
Price (0 = low, 1 = high)	0.212 (0.383)	0.894* (0.375)	−0.141** (0.046)
Payment Method × Price	0.556 (0.542)	−0.055 (0.530)	0.087^†^ (0.046)

^†^*p* < 0.10; **p* < 0.05; ***p* < 0.01; ****p* < 0.001.

TABLE 2 The results of multilevel regressions in Study 2.

**Table 2:**

Study 2			
Dependent Variable	N300	LPP	Purchase Intention
Payment Method	5.283^***^	2.403^***^	0.586^***^
(0 = cash, 1 = mobile payment)	(0.571)	(0.675)	(0.039)
Product Category	0.109	0.821	0.102**
(0 = hedonic, 1 = utility)	(0.572)	(0.675)	(0.039)
Payment Method × Price	−1.127	−1.506	0.077
	(0.809)	(0.954)	(0.056)

^†^*p* < 0.10; **p* < 0.05; ***p* < 0.01; ****p* < 0.001.
